# ACSL3 Promotes Hepatocellular Carcinoma Tumorigenesis and Correlates With JAK–STAT3 Signaling

**DOI:** 10.1002/cam4.71543

**Published:** 2026-02-10

**Authors:** Melika Amelimojarad, Mandana Amelimojarad, Alireza Pourmahdian, Zhang Lu

**Affiliations:** ^1^ Department of Life Science, School of Medicine Iran University of Medical Science Tehran Iran; ^2^ Department of Life Sciences Liaoning University Shenyang China

**Keywords:** ACSL3, LDs, lipid metabolism, liver cancer

## Abstract

**Background:**

Hepatocellular carcinoma (HCC) is a leading cause of cancer mortality with limited therapies. Reprogrammed lipid metabolism, driven by upregulated de novo lipogenesis, is a key tumorigenic mechanism. The enzyme ACSL3 is strongly correlated with poor HCC prognosis, positioning it as a potential therapeutic target.

**Material and Methods:**

ACSL3 expression was assessed in vivo and ex vivo using comparative analysis. Bioinformatic investigations, including gene set enrichment analysis (GSEA) and KEGG pathway analysis, were employed to identify signaling pathways and biological processes associated with ACSL3 overexpression.

**Results:**

ACSL3 expression was consistently elevated in HCC models. Enrichment analyses revealed that high ACSL3 levels are associated with activation of the STAT3 signaling pathway and upregulation of key lipogenic enzymes, suggesting a feedforward oncogenic loop. Predictive data also indicate a correlation between ACSL3 expression and the immune checkpoint regulator PD‐L1.

**Conclusion:**

These findings underscore ACSL3 as a significant biomarker and candidate therapeutic target in HCC. Its role bridges dysregulated lipid metabolism with oncogenic signaling and immune evasion, warranting further investigation into ACSL3‐targeted strategies.

## 1 Introduction

As the third most frequent cause of global cancer mortality in 2022, hepatocellular carcinoma (HCC) represents the most common type of primary liver cancer, comprising 75%–85% of cases [1]. The incidence of HCC is gradually rising in developing countries, becoming the main concern for public health [2]. Regardless of significant advancements in HCC treatment over the previous few decades, including multiple therapies, the majority of patients eventually experience disease progression [3]. Recently, abnormal activation of mitochondrial β‐oxidation and fatty acid metabolism has been considered the new hallmarks in cancers [4, 5]. Accumulating evidence shows that deregulation of fatty acids (FAs) metabolism, including the enhanced de novo FA synthesis, is associated with the development of cancers and affects the tumor microenvironment (TME), such as immune cells, blood vessels, stromal cells, tumor cells, and molecular signaling networks [6, 7]. Therefore, determining the primary elements controlling aberrant lipid metabolism would have a wide variety of effects, from the comprehension of pathogenic pathways to the creation of novel therapeutic targets for efficient cancer treatment [8, 9].

At the moment, the rapid advancements in sequencing technology have made it possible to discover important molecular characteristics that contribute to the development of HCC, through the evaluation of large‐scale, multidirectional data to support more individualized and dynamic treatment plans to manage HCC [10]. The long‐chain acyl‐CoA synthetases (ACSLs) with chain lengths of 12–20 carbon are key players in lipid metabolism due to their roles in catalyzing the transformation of fatty acids (FAs) with long chain to fatty acyl‐CoA [11, 12]. In mammals, ACSLs are made of five isozymes (ACSL1 and ACSL3‐6) [13]. The genomics, protein structures, and catalytic activities of each ACSL family member vary across a wide range of tissues [13].

These variations might affect how FAs are metabolized in various tissues and metabolic circumstances [14, 15]. Importantly, most of the ACSLs are dysregulated in tumors, affecting the species, amount, and distribution of intracellular FAs, which aggravates cancer and other metabolic diseases like obesity, atherosclerosis, and diabetes [16, 17]. As one member of the ACSL family, ACSL3 has been reported to influence metabolic reprogramming in different cancers through multiple interconnected metabolic pathways [18]. Previous studies indicate the essential role of ACSL3 in inducing lipid droplet (LDs) formation in clear cell renal cell carcinoma (ccRCC), while its inhibition reduced lipid accumulation and tumorigenesis [19, 20]. Considering that the exact role of ACSL3 in HCC development remains unknown, therefore, this study aims to discover the impact of altered ACSL3 on HCC tumorigenesis by evaluating its involvement in FAs synthesis and lipid metabolism with the help of bioinformatics through examining the TCGA‐LIHC dataset and GEO datasets, as well as in vitro/in vivo experiments using HCC cell lines to open avenues for novel approaches in HCC treatment.

## 2 Materials and Methods

### 2.1 Cell Culture and Treatment

HCC cell lines, including HepG2 and HUH7, and the normal liver cell line L0‐2 were purchased from the China Academy of Science Co. Ltd. All cell lines were authenticated by an expert before use and verified to be free of mycoplasma contamination. The complete growth medium was prepared by supplementing DMEM or RPMI with 10% fetal bovine serum (FBS) and 1% penicillin/streptomycin under sterile conditions. For cell thawing and seeding, frozen cells were retrieved from liquid nitrogen using protective gloves, rapidly thawed in a 37°C water bath, and sterilized with 75% ethanol. For cell resuspension, cells were transferred to a 15 mL centrifuge tube, centrifuged (1000 rpm, 5 min), and the supernatant was discarded. The cell suspension was added to a 10 cm culture dish containing 8 mL pre‐warmed medium, gently swirled for even distribution, and incubated at 37°C with 5% CO_2_. For cell Treatment and Passage, HCC cells were divided into two groups: untreated controls and siRNA‐treated or vector‐transfected controls, and vector‐transfected transfection. For cell passaging, at 80% confluence, cells were washed with PBS, dissociated with 0.25% trypsin (3–5 min, 37°C), and neutralized with fresh medium. The suspension was centrifuged (1000 rpm, 5 min), and the pellet was resuspended in fresh medium for reseeding. For medium replacement, and cryopreservation medium was replaced every 24–48 h with fresh DMEM/10% FBS after aspirating spent medium [19].

### 2.2 Extraction and Validation of DEGs

The TCGA‐LIHC, and two microarray databases including the (GSE62232 on platform GPL570, with 81 HCC samples and 10 normal samples), and (GSE14520 dataset on GPL571 the consisting of 247 HCC tissues and 241 normal samples) were extracted from the GEO (Gene Expression Omnibus) database. Genes with low levels of expression and low variance across samples were eliminated before the creation of gene co‐expression networks. Next, the “limma” package from R software (version 4.3.1) was used to normalize the microarray dataset [21], Adjusted *p*‐value (FDR) < 0.05 and |logFC| ≥ 1 [22]. The expression of selected genes was also evaluated in HCC tumor tissues and normal tissues using UALCAN and GEPIA2 database (http://gepia2.cancer‐pku.cn/#analysis). The relationship between expressions of our selected gene with clinical data, such as age, and different pathological stages, was also extracted from http://ualcan.path.uab.edu. The association between our selected gene and drug sensitivity was extracted from the GSCA database (https://guolab.wchscu.cn/GSCA/). The overall survival (OS), the “survival analysis,” was extracted from KM plotter software, and the Human Protein Atlas (HPA) (https://www.proteinatlas.org) was used to obtain immunohistochemistry images of our final gene between HCC and normal tissues. The gene enrichment analysis (GSEA) was detected using Cluster Profiler, in which the normalized enrichment scores (NES) were calculated for each gene set. Statistical significance was set at |NES| > 1, nominal *p* value < 0.05, and FDR *q*‐value < 0.25.

### 2.3 RNA Extraction and q‐PCR

#### 2.3.1 For qRT‐PCR Analysis

RNA Extraction: Total RNA was isolated using a commercial kit, and purity/concentration was assessed via a nanodrop spectrophotometer. For cDNA synthesis, RNA (1 μg) was reverse‐transcribed using a thermal cycler under the following conditions: 27°C (15 min), 37°C (45 min), and 85°C (5 min). Quantitative PCR: Reactions contained SYBR Green mix, primers, dNTPs, and cDNA templates. Amplification was performed on a 7500 Real‐Time PCR System (Applied Biosystems). β‐actin served as the internal control. Relative mRNA expression was calculated using the 2^−ΔΔCt^ method. Primer sequences are listed in Table 1.

**TABLE 1:** List of primers for qRT‐PCR.

Genes name	Sequences
ACSL3 F sequence	5‐ACCAGGGAAATCCTAAGTGAAG‐3
ACSL3 R sequence	5‐GGTGTTCTTTGGTTTTAGTCCC‐3
β‐Actin F sequence	5‐AGGTCTTGCGGATGACCACGT‐3
β‐Actin R sequence	5‐GTCCACCACCCTGTTGCTGTAG‐3

### 2.4 Cell Transfection

The human HCC cell line (HepG2, HUH7) was cultured in complete DMEM including penicillin, streptomycin, and 10% fetal bovine serum, incubated in a 5% CO_2_ incubator. For transfection, two different pre‐designed si‐ACSL3 (10 μM), the si‐control (Ambion) (catalog number 4618G) as shown in Table 2. The 1 μg/mol pcDNA3.1(+) ACSL3 vector and empty vector were acquired from Gene Pharma, Shanghai, China, to alter ACSL3 expression in HCC cells by the Lipofectamine 8000 reagent (Keygen Biotech, China) in accordance with the manufacturer's instructions.

**TABLE 2:** List of si‐RNAs for ACSL3 gene knockdown.

Gene name	Sequences
si‐control	5‐UGGUUUACAUGUUUUCCUA‐3
siACSL3	5‐UAACUGAACUAGCUCGAAA‐3
siACSL3*	5‐GCAGUAAUCAUGUACACAA‐3

### 2.5 Cell Viability Estimation

Cell viability was estimated using a Cell Counting Kit‐8 (CCK‐8) kit (Beyotime, China). Briefly, the HepG2 and HUH7 cells in healthy and good condition were counted under a microscope after being prepared into a cell suspension using standard cell passage procedures. 4000–6000 cells per well were seeded into a sterile 96‐well plate at a density of 5 × 10^4^ cells/well and were cultured overnight at 37°C with 5% CO_2_ under sterile conditions. Treatments are administered to the cells based on their pre‐grouping once they reach 60%–70% growth, with at least five repetitions for each group. Once the culture has reached the predetermined dosing time, remove the old culture solution and add 10 μL of CCK‐8 solution and 100 μL of serum‐free medium to each well and measure the absorbance at 450 nm with a microplate reader (Model 680 microplate reader, Bio‐Rad Laboratories).

### 2.6 Colony Formation

The fully grown HCC cells were treated with our inhibitor (siRNA) or overexpression vector, left to grow overnight, and the next day, the medium was changed and incubated for two weeks. Then the crystal violet 1% solution in ethanol was used for coloring the HCC cell colonies after cell fixation with formalin 10%. Finally, ImageJ software was used to document images, count colonies, and plot graphs using GraphPad Prism [23].

### 2.7 Western Blot Examination

We start the process by protein extraction using pre‐chilled RIPA lysis buffer, sample preparation, and quantification. The BCA working solution was then prepared by mixing reagents A and B (50:1 ratio), and the absorbance was measured at 562 nm using a plate reader, and protein concentration was determined by comparing the sample absorbance to a generated standard curve.

After the SDS‐PAGE gel run of protein samples, we activated the PVDF membrane with methanol for a minute beforehand before being immersed in the membrane transfer solution with the filter paper.

After blocking the membranes for 2 h in skim milk 5%, and the first antibody ACSL3(1:1000), STAT3/p‐STAT3(1:1500), JAK/p‐JAK (1:1500), and beta‐actin (1:1000), respectively, overnight at 4°C, membranes underwent three 10‐min washes with 1 × TBST before being incubated with secondary antibody (1:2000) for 2 h at room temperature. Place the PVDF film on a chemiluminescent tray after thoroughly mixing the ECL working solution in a 1:1 ratio and draining any excess liquid. The density of specific bands was estimated using ImageJ image analysis software. Observe the development scenario and calculate the exposure duration based on protein expression. Store the PVDF membrane 1 × Clean TBST in a refrigerator at −20°C after three cleanings.

### 2.8 Correlation Analysis of Immune Markers

We evaluate the relationship between high ACSL3 and immune cell infiltration using the TIMER tool(“https://cistrome.shinyapps.io/timer/), and immune cell data using the CAMOIP (www.camoip.net) web tool, which facilitates analyzing the biological pathways and mechanisms involved in these genes using the CIBERSORT algorithm and TNM plot.

### 2.9 Lipid Accumulation and Intracellular TG Analysis

TG content was determined enzymatically using a TG assay kit (Solarbio, China) exactly following the instructions on the kit, and the concentration of TG was evaluated at 520 nm. The intracellular lipid was assessed by using red oil staining on FFA‐treated HepG2 cells with a palmitic acid: oleic: 2 ratios. PBS (phosphate‐buffered saline) was used to wash the cells before they were fixed for 4% paraformaldehyde. Following fixation, cells were stained for 60 min at room temperature using Oil‐Red‐O solution, followed by a PBS wash to remove any unbound stain. The absorbance of the solution was then measured at 490–520 nm.

### 2.10 HCC Xenograft Mouse Model Designs

Six‐week‐old male BALB/c nude mice were obtained from Liaoning Changsheng Biotechnology Co. All mice were housed under specific pathogen‐free (SPF) conditions with a 12‐h light/dark cycle and provided with autoclaved food and water ad libitum. The study protocol was reviewed and approved by the Institutional Animal Care and Use Committee (IACUC) of Liaoyang University (Approval Code: AE18055), and all procedures were conducted in strict accordance with the committee's ethical guidelines. After 1 week of acclimatization, HUH7 hepatocellular carcinoma cells were harvested and resuspended in a 1:1 mixture of serum‐free medium and Matrigel. Each mouse received a single subcutaneous injection of 100 μL of this suspension, containing 5 × 10^5^ cells, into the right flank. Mice were monitored daily, and tumor growth was measured using digital calipers. When the average tumor volume reached approximately 50–100 mm^3^ (palpable size), the mice were randomly divided into two experimental groups (*n* = 4 per group). Treatment was administered via intravenous tail vein injection every 3 days for a total of 5 injections over 15 days. The siRNA dose was [2 mg/kg] CAT#: NRYF‐0625‐XY1384. Tumor volume and body weight were measured every other day. Tumor volumes (in mm^3^) were calculated using the formula: Volume = (Long Diameter × Short Diameter^2^)/2.

### 2.11 Statistical Analysis

For microarray workflows, pre‐processing, including background correction, log_2_ transformation, and quantile normalization, was conducted on raw expression data to ensure comparability across samples. Outliers were identified using PCA/boxplots and removed if necessary, and batch effects were corrected using standard algorithms such as ComBat to minimize non‐biological variation. All statistical analyses were performed using GraphPad Prism version 9.0 (GraphPad Software, San Diego, CA, USA). Data were first examined for normality and variance homogeneity to ensure the validity of parametric testing. Comparisons among multiple groups were conducted using one‐way analysis of variance (ANOVA) followed, when appropriate, by post hoc Student's *t*‐tests. A two‐tailed *p* value < 0.05 was considered statistically significant. Quantitative data are presented as mean ± standard deviation (SD) and the sample size (*n*) for each experiment is indicated in the corresponding figure legends. All experiments were independently repeated at least three times.

## 3 Results

### 3.1 Identification of Potential HCC DEGs

**FIGURE 1:**
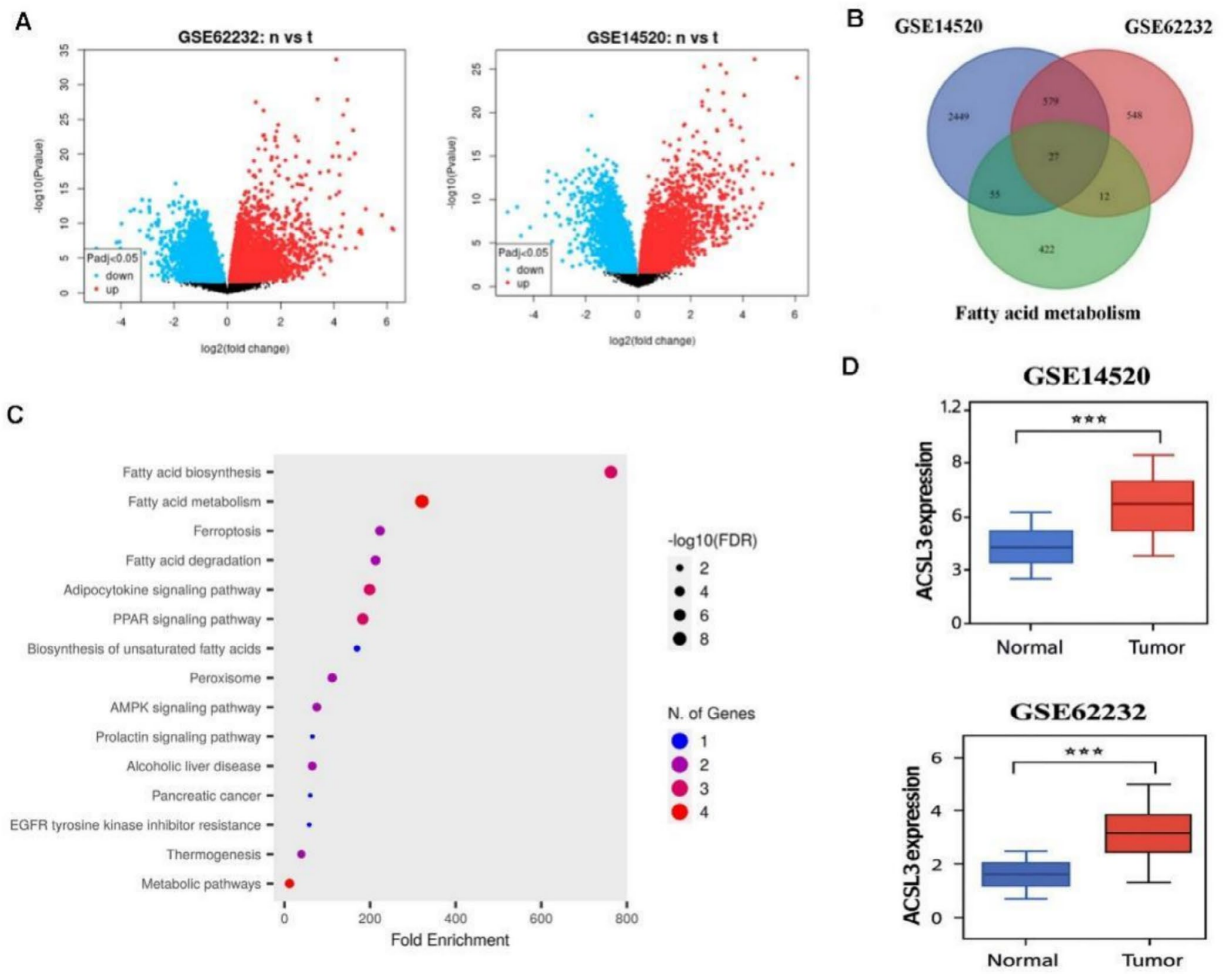
Identification and characterization of potential HCC‐associated differentially expressed genes (DEGs). (A) Volcano plots of HCC versus non‐tumor liver tissues from the GSE14520 and GSE62232 datasets, showing significantly upregulated genes (red) and downregulated genes (blue). Vertical lines indicate the log_2_ fold‐change threshold, and the horizontal line denotes the statistical significance cutoff. (B) Venn diagram illustrating the overlap of DEGs between the GSE14520 and GSE62232 datasets. (C) KEGG pathway enrichment analysis of the shared DEGs. (D) Box plots showing significantly elevated ACSL3 mRNA expression in tumor tissues compared with non‐tumor controls across both datasets (*n* = 536 total samples). Statistical significance was assessed using a student's *t*‐test (***p < 0.001).

The volcano plot visualization of differently expressed genes (DEGs) detected among two different human HCC tissues microarrays, GSE14520 (225 HCC samples and 220 non‐cancer samples) and GSE62232 (81 HCC samples and 10 normal liver samples), using |logFC| > 1 and adjusted *p* < 0.05 criteria using the R software program [22]. A Venn diagram determined the overlapping DEGs between two GSEs and lipid metabolism hallmarks, and their involvement in biological pathways was evaluated using KEGG enrichment analysis, indicating their involvement in fatty acid metabolism and the RRAR signaling pathway. The ACSL3 expression was one of the overlapping genes among both GSEs, and its expression was highly evaluated in HCC tissues compared to normal tissues (Figure 1A–D).

### 3.2 High ACSL3 Is Negatively Associated With Patients' Clinical Outcomes

**FIGURE 2:**
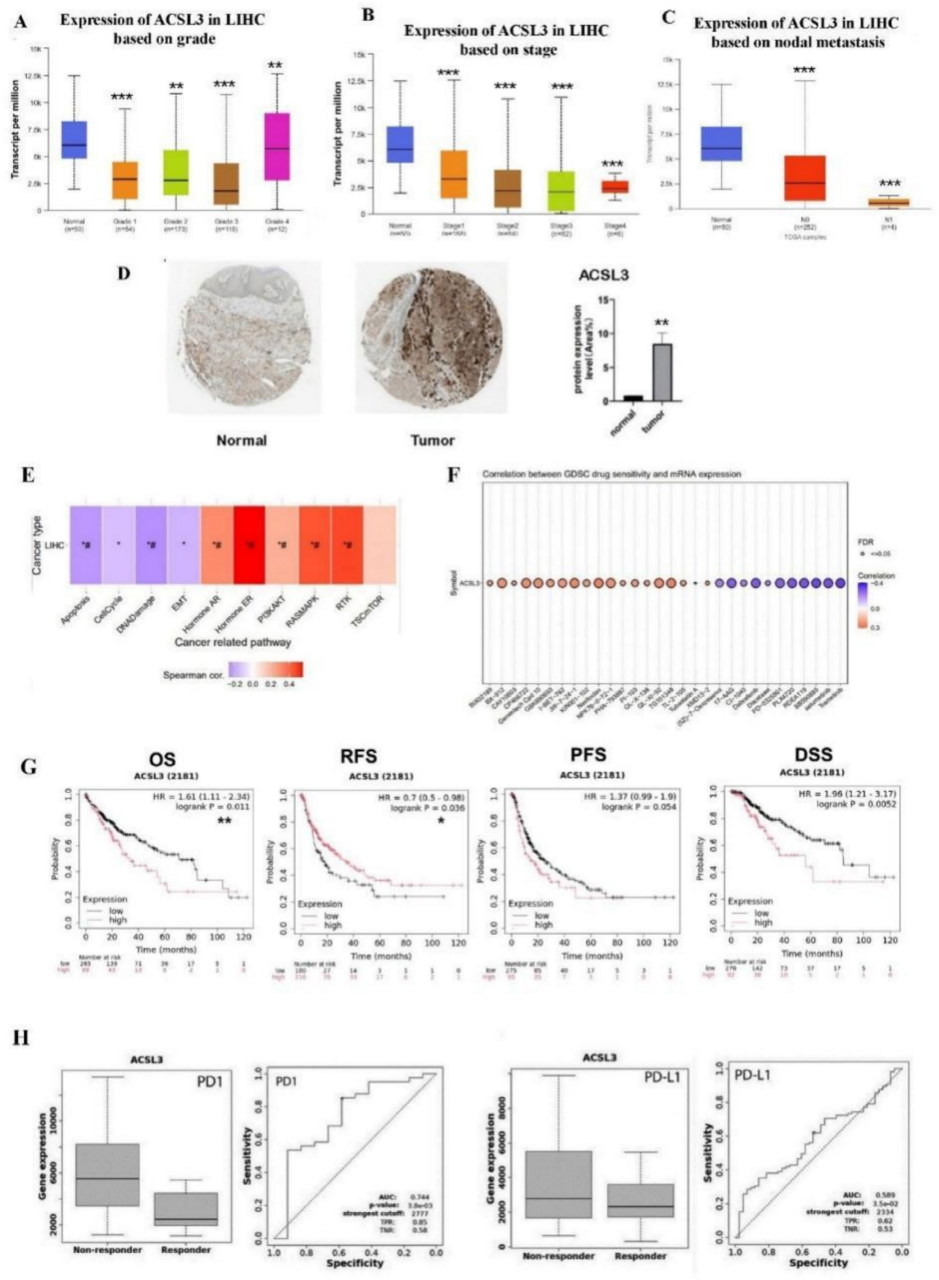
The Negative Prognostic Impact of ACSL3 in HCC. (A‐C) Association between high ACSL3 mRNA expression and advanced clinicopathological features in the TCGA‐LIHC cohort. High ACSL3 expression was significantly correlated with (A) higher tumor stage, (B) higher tumor grade, and (C) the presence of nodal metastasis. Data are presented as box plots showing the median, quartiles, and distribution of expression levels (D). Representative immunohistochemistry (IHC) images from the Human Protein Atlas (HPA) database confirming elevated ACSL3 protein expression in human HCC tissue compared to adjacent normal liver tissue. Scale bar, 100 μm. (E) Gene Set Enrichment Analysis (GSEA) reveals cancer‐related pathways significantly enriched in tumors with high ACSL3 expression. The plot shows the normalized enrichment score (NES) and false discovery rate (FDR) for the top‐associated Hallmark pathways. (F) Drug sensitivity analysis illustrating the correlation between high ACSL3 expression and increased resistance or sensitivity to specific chemotherapeutic agents. (G) Kaplan–Meier survival curves demonstrating the significant impact of high ACSL3 expression on reduced patient survival. High expression is associated with poorer (i) Overall Survival (OS) and (ii) Recurrence‐Free Survival (RFS) (Log‐rank test, *p* < 0.01). (H) Analysis of immunotherapy response showing that high ACSL3 expression may be associated with a lack of response to PD‐1/PD‐L1 checkpoint inhibitors in a cohort of treated HCC patients. The bar plot compares the rate of non‐responders between high and low ACSL3 expression groups (*p* < 0.05). Data are presented as mean ± SEM. The TCGA‐LIHC cohort includes 427 samples (377 tumor and 50 normal). Statistical significance was assessed using a student's *t*‐test or one‐way ANOVA for panels A–C, and a Chi‐squared test for panel H (*p < 0.05; **p < 0.01; ***p < 0.001).

The influence of highly expressed ACSL3 on patient characteristics and clinical outcomes in HCC tissues was evaluated from UALCAN, GEPIA2, and GSCA databases. Based on our results, high ACSL3 expression could negatively affect the patients' clinical outcomes, such as tumor staging, nodal metastases, as well as patients' prognosis (OS, RS). Immunome staining data of patients also confirmed the high ACSL3 protein levels in HCC tumor tissues compared to normal tissues. In addition, the negative connection of elevated ACSL3 with different biological pathways, such as PI3K/AKT and hormone ER, has been detected in HCC. The correlation between drug sensitivity and ACSL3 overexpression suggests that high ACSL3 expression can influence the reaction of various anti‐cancer medications in patients. Next, we have evaluated the effect of highly expressed ACSL3 on cancer immune checkpoint inhibitors (ICIs) treatment response (anti‐PD‐L1 therapy and anti‐PD1) by dividing the data into two groups: responders and non‐responders. Our data showed that in all samples, increased ACSL3 is estimated to be strongly correlated with treatment resistance, which suggests that ACSL3 could be a potential target for overcoming chemotherapy resistance in HCC treatment (Figure 2A–H).

### 3.3 The Impact of High ACSL3 Expression on Immune Filtration

**FIGURE 3:**
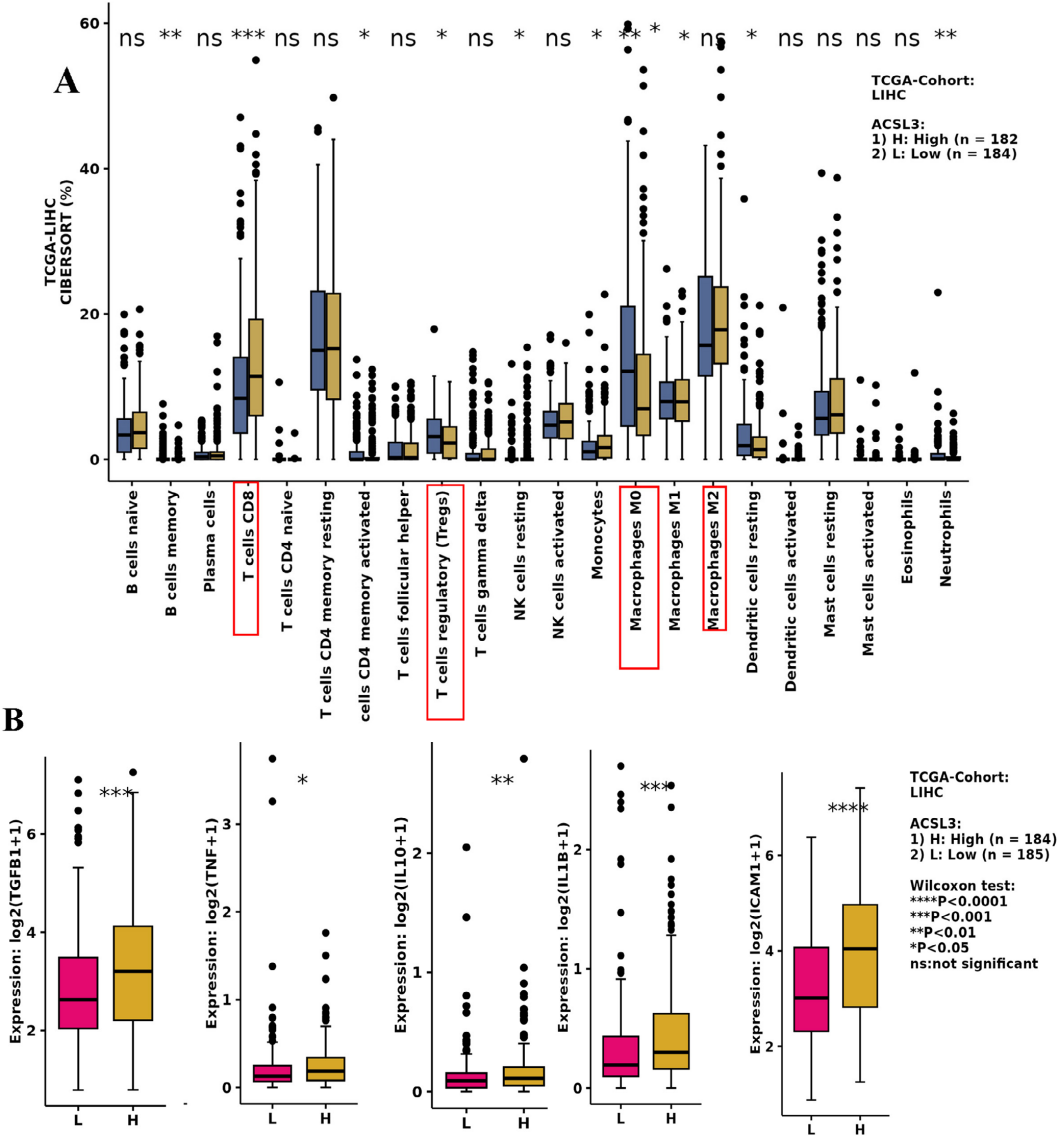
Association of High ACSL3 Expression with Altered Immune Infiltration in the Tumor Microenvironment (TME) of HCC. (A) Violin plots (or box plots) demonstrating the correlation between high ACSL3 expression and the relative abundance of specific tumor‐infiltrating immune cells, as estimated by CIBERSORTx analysis of the TCGA‐LIHC transcriptomic data, *n* = 366. High ACSL3 expression was significantly associated with decreased infiltration of CD8+ T cells and increased infiltration of macrophages and regulatory T cells (Tregs). (B) Analysis of inflammatory cytokine expression reveals that high ACSL3 expression is significantly correlated with elevated levels of IL‐10 in the HCC TME, suggesting a role for ACSL3 in promoting an immunosuppressive microenvironment. Statistical significance was determined using Pearson's (or Spearman's) correlation test, *n* = 369. Data are presented as mean ± SEM (*p < 0.05; **p < 0.01; ***p < 0.001).

Given the critical role of ACSL3 in lipid synthesis and lipid droplet (LD) accumulation, and emerging evidence linking lipid metabolic reprogramming to tumor immune microenvironment (TME) dynamics [21], we investigated how ACSL3 overexpression influences immune cell composition and anti‐tumor responses in HCC by using CIBERSORT algorithm analysis of transcriptomic data from 369 HCC patients, we observed that ACSL3‐driven lipid accumulation correlates with altered immune cell populations. Notably, tumors with elevated ACSL3 expression exhibited a significant increase in CD8+ T‐cell infiltration. These cytotoxic T cells may actively shape lipid metabolism within the TME by releasing inflammatory cytokines (e.g., IL‐10, TNF‐α), which in turn upregulate lipid synthesis genes. This feedforward loop enhances fatty acid (FA) availability, potentially fueling HCC progression by providing lipid substrates for membrane biosynthesis and energy demands of proliferating tumor cells (Figure 3A,B).

### 3.4 High ACSL3 Is Connected With Lipid Metabolism Regulatory Genes and Pathways

**FIGURE 4:**
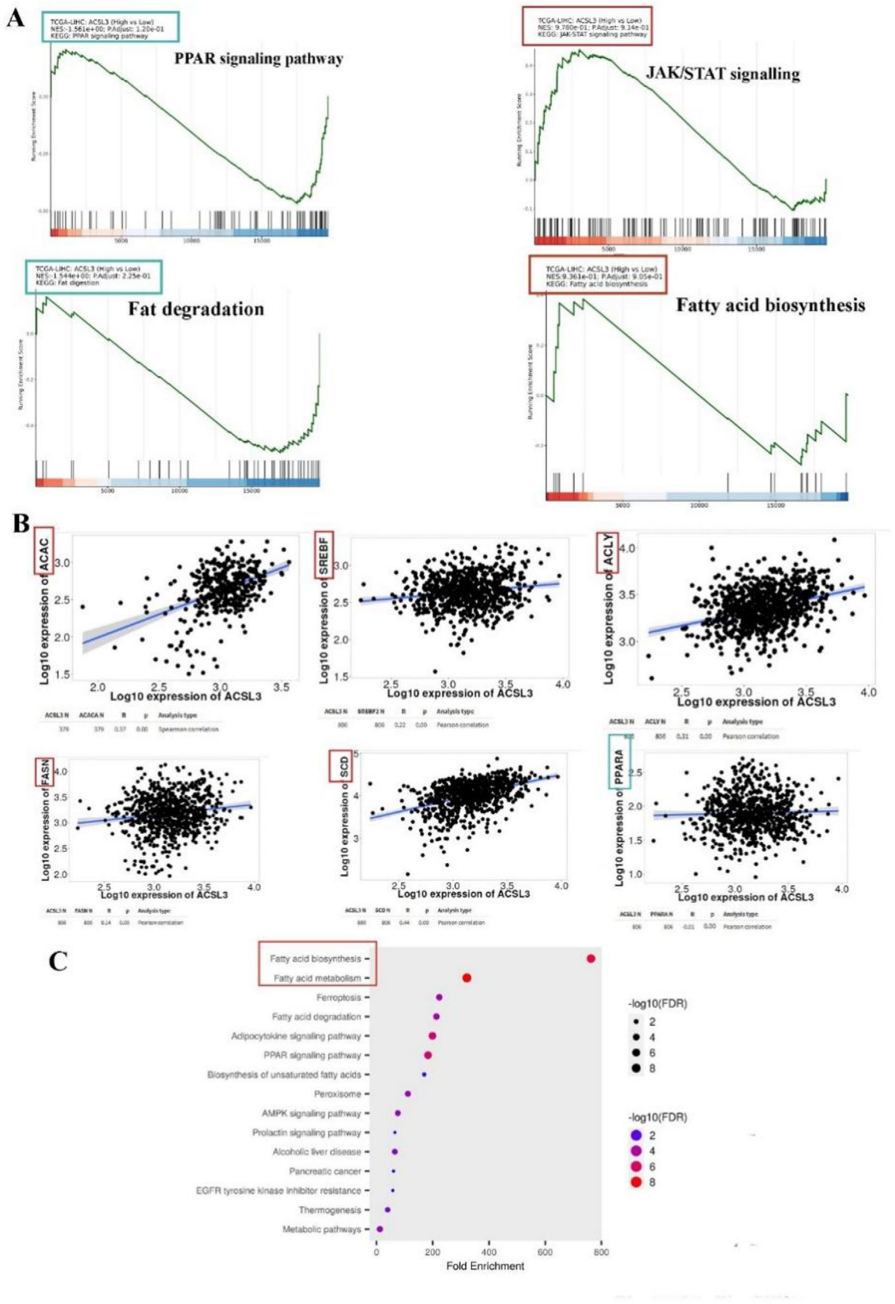
The Impact of High ACSL3 on Lipid Metabolism Pathways and Biological Processes in HCC. (A) Gene Set Enrichment Analysis (GSEA) plot showing a significant positive enrichment of the JAK–STAT signaling pathway in tumors with high ACSL3 expression, linking ACSL3 to key signaling events in HCC metabolic reprogramming. The Normalized Enrichment Score (NES) and false discovery rate (FDR) are indicated. (B) Scatter plots showing a significant positive correlation between ACSL3 mRNA expression and key de novo lipogenesis (DNL) enzymes (FASN, ACC, and SCD) in the TCGA‐LIHC cohort, as determined by Pearson's correlation analysis. This suggests a coordinated role in promoting lipid accumulation. (C) Functional enrichment analysis of genes co‐expressed with ACSL3. The bar chart displays significantly enriched Gene Ontology (GO) terms for biological processes, colored by the average log2 fold change (logFC) of associated genes, indicating the direction and strength of the association. For GSEA in (A), an NES > 1.5 and an FDR *q*‐value < 0.25 were considered evidence of significant enrichment, *n* = 366.

To investigate how ACSL3 supports lipid metabolism reprogramming in HCC, we performed GSEA and KEGG pathway analysis based on ACSL3 expression levels. Tumors with high ACSL3 expression showed significant enrichment in the JAK/STAT3 pathway, a key regulator of de novo lipogenesis (DNL). Mechanistically, STAT3 activation transcriptionally upregulates critical lipogenic enzymes, including fatty acid synthase (FASN), stearoyl‐CoA desaturase (SCD), and acetyl‐CoA carboxylase (ACAC), which collectively drive fatty acid synthesis to fuel HCC growth [22]. Notably, ACSL3 overexpression also correlated with suppression of fatty acid degradation pathways, including reduced PPAR signaling activity. This dual effect (enhanced DNL and impaired degradation) promotes lipid droplet (LD) accumulation and triglyceride (TG) synthesis, creating a lipid‐rich microenvironment conducive to tumor progression. Together, these findings position ACSL3 as a central node linking STAT3‐driven lipogenesis and metabolic reprogramming in HCC, highlighting its potential as a therapeutic target to disrupt lipid metabolism dependencies in HCC (Figure 4A–C).

### 3.5 ACSL3 Is Significantly Increased in HCC Tissues and Cell Lines

**FIGURE 5:**
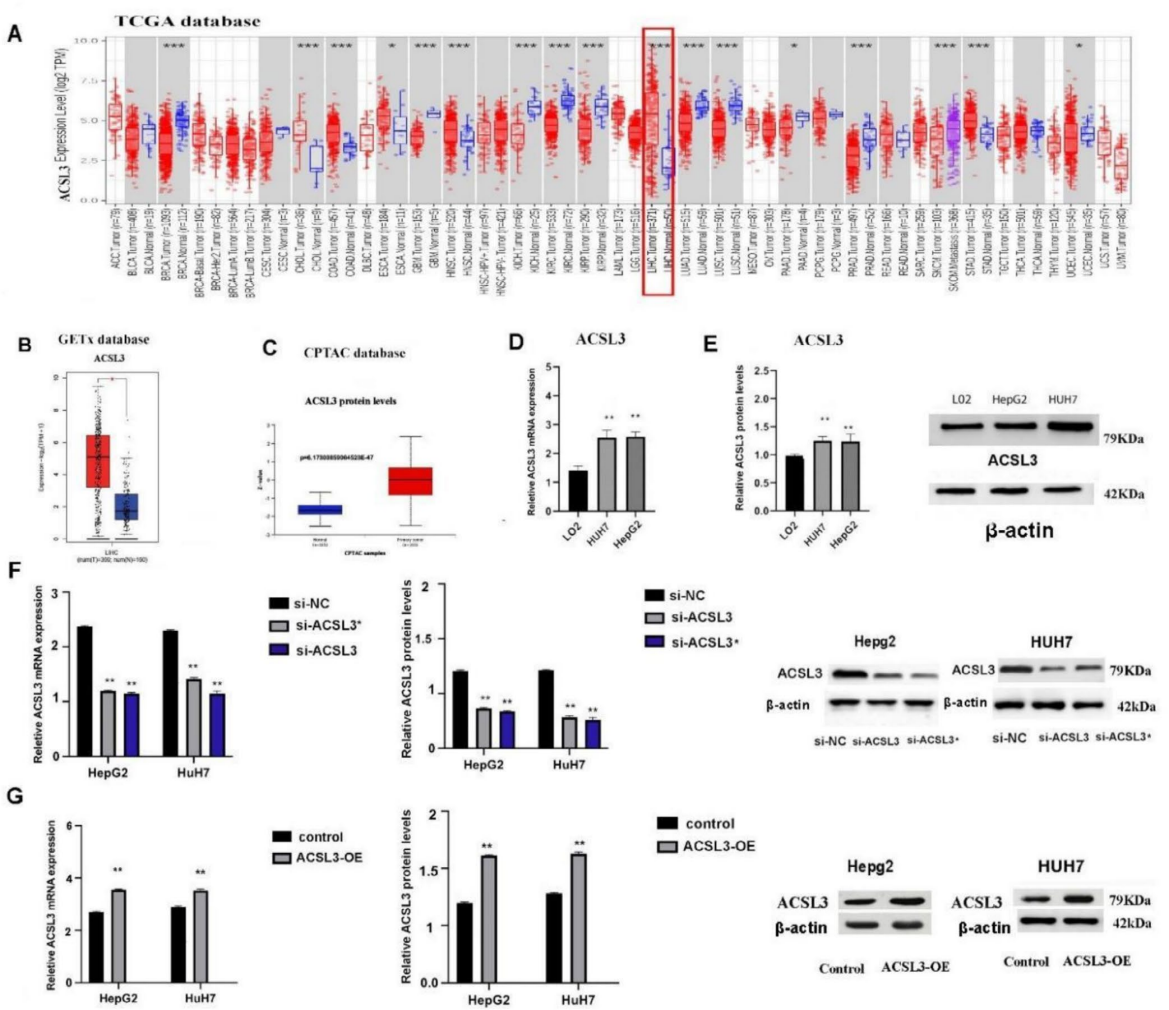
ACSL3 is highly expressed in HCC and successfully targeted for functional studies. (A) Pan‐cancer analysis of ACSL3 mRNA expression in tumor tissues compared to paired normal tissues from the Linked Omics database (*n* = 17 cancer types). (B, C) Analysis of ACSL3 (B) mRNA and (C) protein expression levels in HCC versus normal liver tissues from online repositories (TCGA, CPTAC). (D, E) Validation of high ACSL3 (D) mRNA and (E) protein expression in human HCC cell lines (HepG2, Huh7) compared to the normal human hepatocyte cell line (L‐02). (F) Efficiency of ACSL3 knockdown using two different siRNAs (si‐ACSL3#1, si‐ACSL3#2) as measured by RT‐qPCR and western blot. (G) Efficiency of ACSL3 overexpression (ACSL3‐OE) as measured by RT‐qPCR and western blot. Data are presented as mean ± SD from at least three independent experiments. si‐ACSL3#1 was selected for subsequent experiments based on its high knockdown efficiency. Data represent mean ± SD from *n* = 3 independent experiments, each performed in triplicate using one‐way ANOVA and unpaired *t*‐test. Statistical significance is denoted as **p < 0.01versus the respective control group (unpaired *t*‐test, and one‐way ANOVA).

Through using the TCGA database, the high ACSL3 expression was detected across the 17 different tumors, including Uterine corpus endometrial cancer (UCEC), Colon adenocarcinoma (COAD), and liver hepatocellular carcinoma (LIHC). Subsequently, extracted data from GETx and CPTAC database also indicated the higher ACSL3 expression in HCC tissues. The accuracy of bioinformatic results was confirmed by PCR and WB evaluating the ACSL3 expression both in mRNA and protein levels in HCC cell lines (HepG2 and HUH7) compared to normal liver cell line L0‐2. We also evaluated the impact of ACSL3 gene knock down or over‐expression in HCC cells. Accordingly, realizing that ACSL3 is significantly upregulated in both HCC tissues and cell lines while its gene knock down significantly reduces its expression (Figure 5A–G).

### 3.6 High ACSL3 Can Promote HCC Cells' Proliferation, Invasion, and Metastasis

**FIGURE 6:**
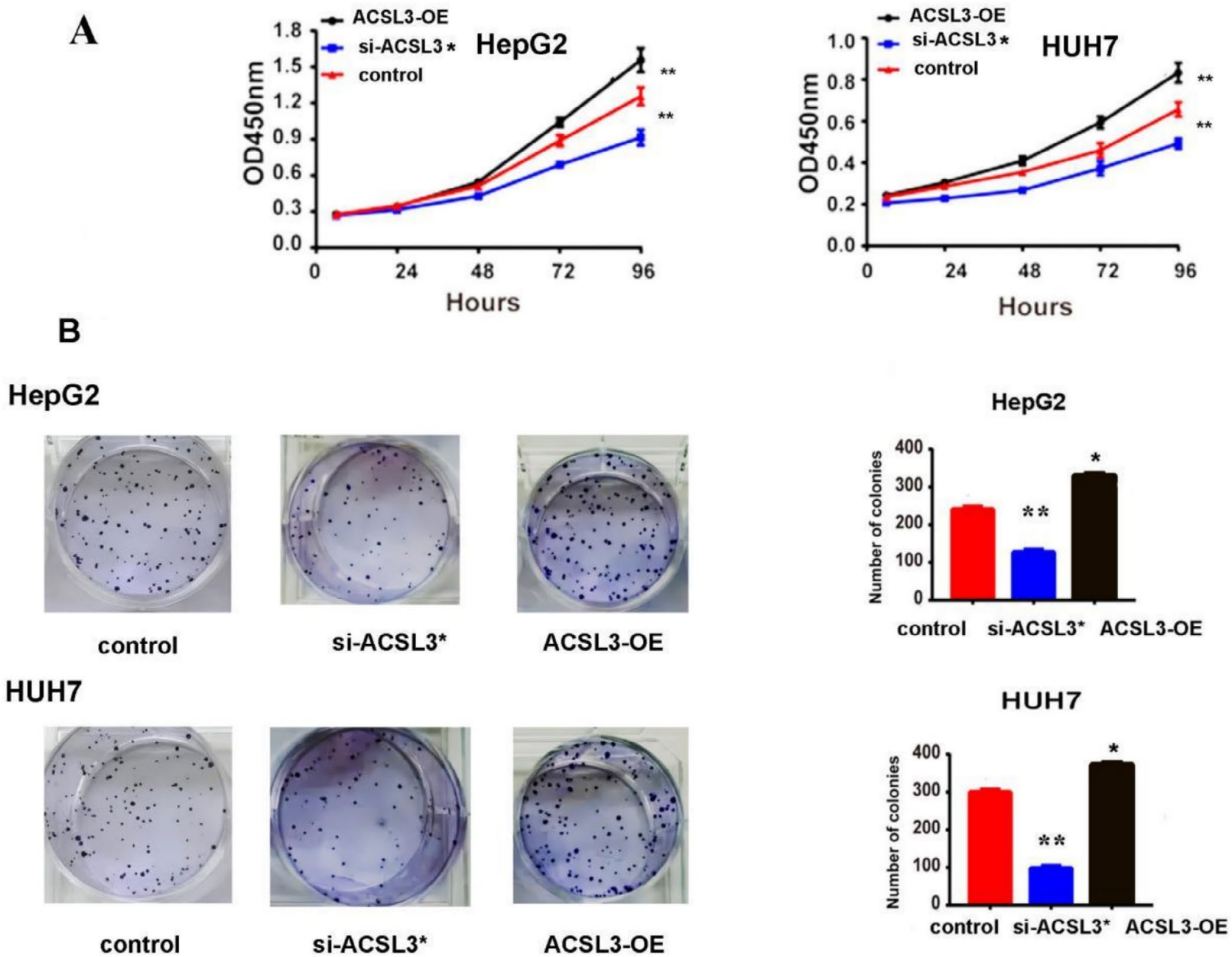
ACSL3 promotes HCC cell proliferation and clonogenicity. (A) Cell viability was assessed by CCK‐8 assay in HepG2 and HUH7 cells following ACSL3 knockdown (si‐ACSL3) or overexpression (ACSL3‐OE) over 72 h. (B) Representative images and quantification of colony formation assays in HepG2 and HUH7 cells after ACSL3 knockdown or overexpression. Data represent mean ± SD from *n* = 3 independent experiments, each performed in triplicate. Statistical significance was determined relative to the respective control group using an unpaired *t*‐test, one‐way ANOVA (*p < 0.05; **p < 0.01).

The functional consequences of ACSL3 expression on HCC malignancy were assessed by manipulating its levels in HepG2 and Huh7 cells. Functional assays revealed that ACSL3 knockdown significantly suppressed cell viability (CCK‐8 assay) and colony‐forming capacity, while ACSL3 overexpression produced the opposite effect, enhancing proliferative and clonogenic potential (Figure 6A,B). These data establish a causal role for ACSL3 in promoting aggressive tumor cell behavior in vitro.

### 3.7 The JAK/STAT3 Signaling Pathway Is Associated With High ACSL3

**FIGURE 7:**
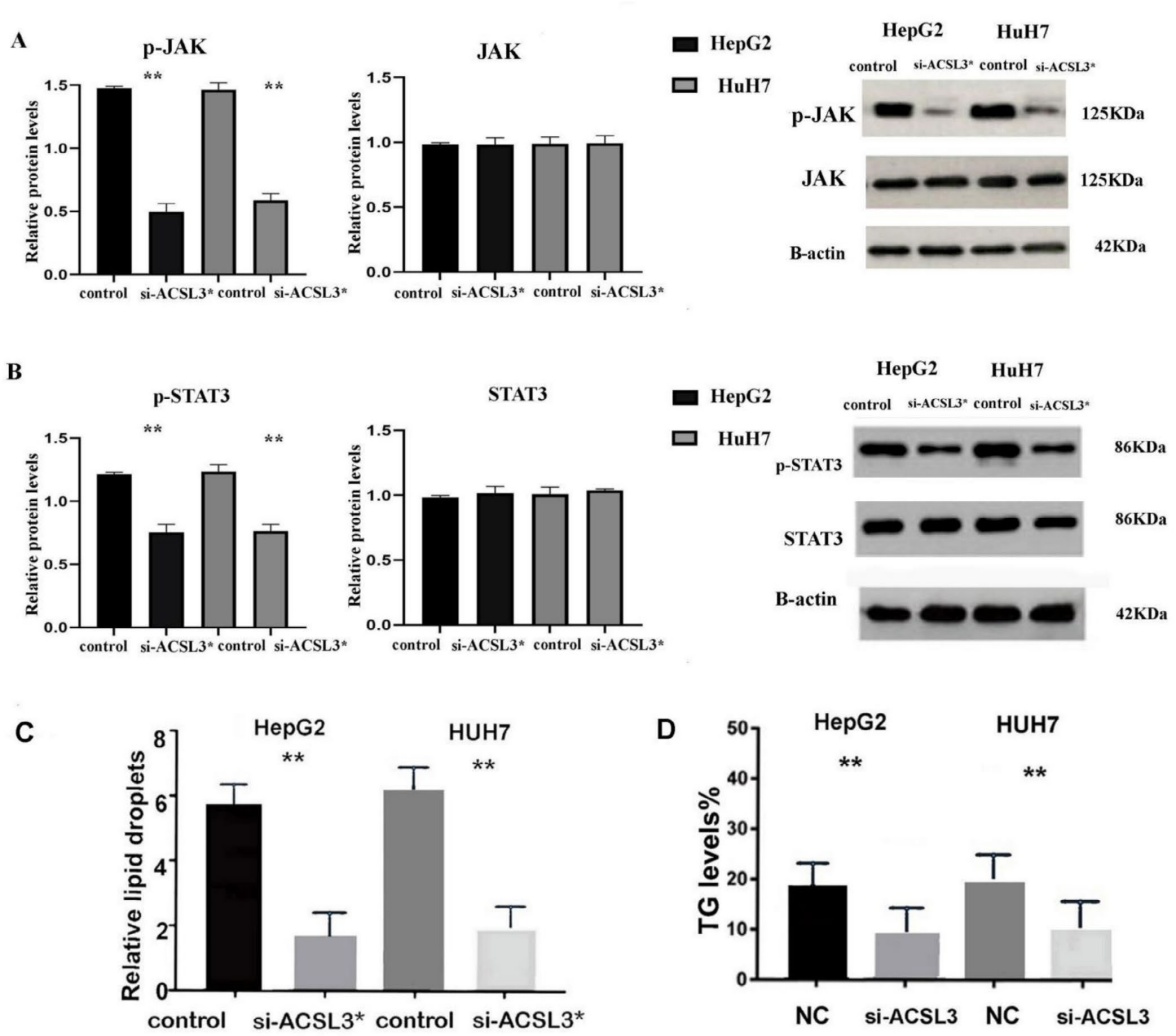
ACSL3 promotes lipogenesis in HCC through activation of the JAK–STAT3 signaling pathway. (A) Scatter plots showing the positive correlation between ACSL3 mRNA expression and JAK1 or STAT3 expression in human HCC samples (TCGA dataset). (B) Western blot analysis demonstrating that ACSL3 knockdown reduces the phosphorylation of JAK and STAT3, indicating suppression of pathway activity. (C) Representative images (scale bar: 100 μm) and quantification of Oil Red O staining showing decreased lipid droplet (LD) accumulation following ACSL3 knockdown (*n* = 3 independent experiments). (D) Quantification of intracellular triglyceride (TG) levels confirms reduced lipid synthesis upon ACSL3 inhibition (*n* = 3 independent experiments). Representative blots are shown from *n* = 3 independent experiments, and densitometric values are presented as mean ± SD. Statistical significance was determined relative to the si‐control group using an unpaired *t*‐test (** *p* < 0.01).

We evaluated the correlation of ACSL3 and STAT3 and JAK1 in HCC tumors based on their roles in regulating metabolic activities using Pearson correlation analysis, confirming the positive correlation, which was in accordance with the GSEA enrichment results, indicating that high ACSL3 is mainly enriched in JAK–STAT signaling in HCC [24] (Figure 7A). We also evaluate the impact of ACSL3 knockdown on the expression of JAK/STAT pathway activity [25]. According to our WB results, ACSL3 inhibition can reduce p‐JAK and p‐STAT3 activity (Figure 7B). Considering the role of ACSL3 in promoting lipogenesis through JAK/STAT3 activity, we further evaluate the role of si‐ACSL3 on LDs accumulation and TGs content, which are intricately linked to cellular energy metabolism, based on the result that ACSL3 gene knockdown could significantly reduce lipid accumulation in HCC cell lines (Figure 7C,D).

### 3.8 siACSL3 Reduced Tumor Growth in the Xenograft Mouse Model

**FIGURE 8:**
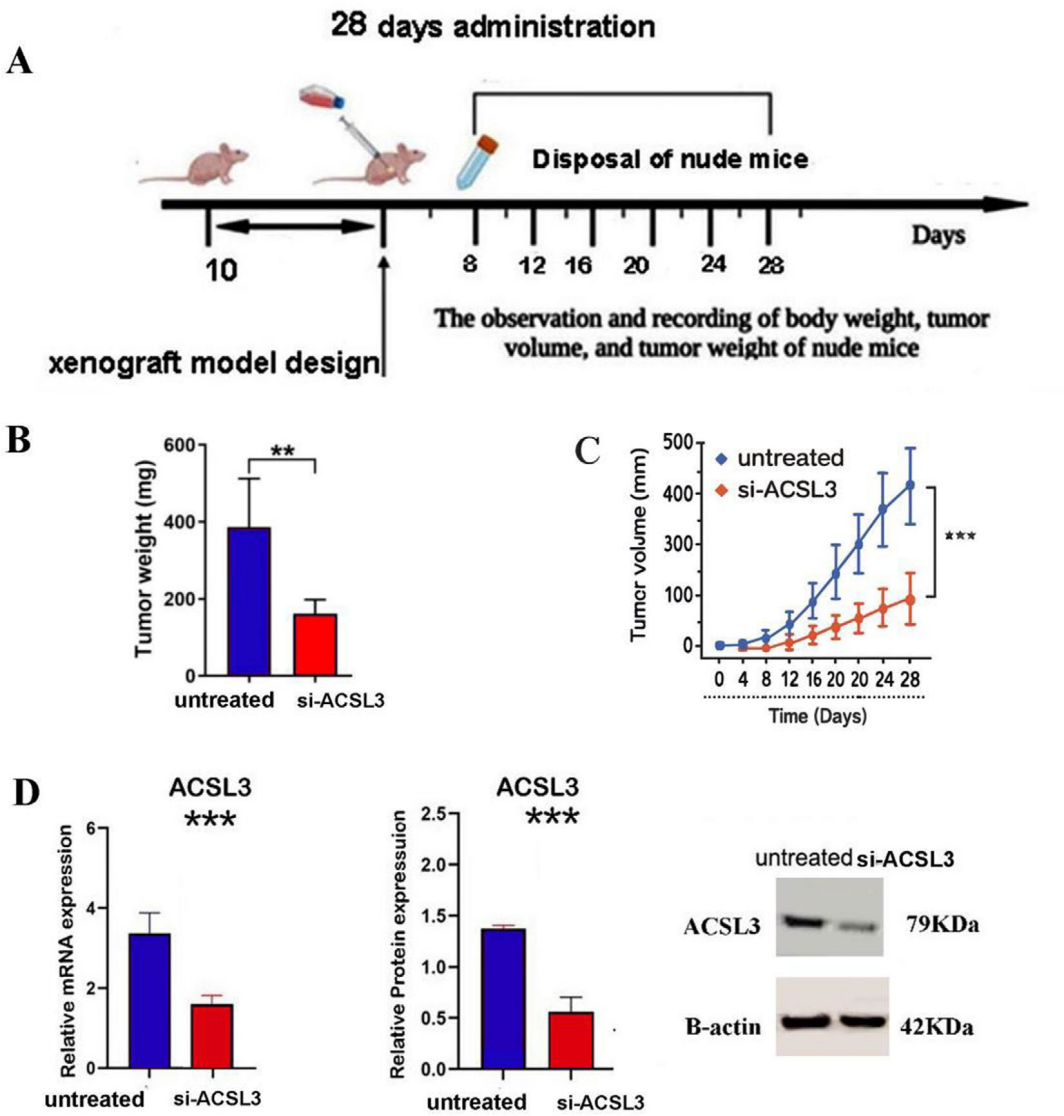
si‐ACSL3 inhibits tumor growth and downregulates ACSL3 expression in a HUH7 xenograft model. (A) Schematic timeline of the experimental procedure, including tumor cell implantation, siRNA treatment schedule, and endpoint analysis. (B) Representative images of excised tumors from control and si‐ACSL3–treated mice at the study endpoint (day 28). (C) Tumor growth curves over time for mice treated with si‐ACSL3 or scrambled control siRNA (*n* = 4 per group). Tumor volume was measured periodically and calculated as (*L* × *W* ^2^)/2. (D) si‐ACSL3 reduces ACSL3 expression in xenograft tumors. Left: Relative ACSL3 mRNA levels quantified by qRT‐PCR. Right: Representative western blot and corresponding densitometric analysis of ACSL3 protein expression. Data are presented as mean ± SD from triplicate measurements, with *n* = 4 mice per group. Statistical significance was determined using an unpaired *t*‐test for panel D and two‐way ANOVA for panel C (**p < 0.01; ***p < 0.001 versus control).

The in vivo antitumor efficacy of si‐ACSL3 was evaluated in the established HUH7 xenograft model. Tumor growth was monitored regularly from day 10 post‐inoculation until the study endpoint at day 28. Mice were randomly assigned to two treatment groups, including the si‐ACSL3 group and the untreated control group (*n* = 4 per group). Tumor volume was measured every 3 days using digital calipers. The volume (*V*) was calculated using the formula: *V* = (*L* × *W* ^2^)/2, where L is the long diameter and W is the short diameter. At the endpoint (day 28), mice were euthanized, and tumors were excised and weighed. The tumor volume and final tumor weight of the si‐ACSL3 group were compared to the control group to determine the treatment's inhibitory effect. To confirm the mechanism of action, the relative expression levels of ACSL3 mRNA and protein in tumor tissues were analyzed. Total RNA and protein were extracted from snap‐frozen tumor samples. Immunoblotting (Western Blot): Protein lysates were separated by SDS‐PAGE, transferred to a membrane, and probed with a primary antibody against ACSL3. Densitometric analysis of the immunoblotting data and quantification of q‐PCR results demonstrated a significant reduction in ACSL3 expression at both the transcriptional and translational levels in the si‐ACSL3 treatment group compared to the control (Figure 8A–D).

## 4 Discussion

The current therapeutic landscape for advanced HCC is dominated by immunotherapies and multi‐kinase inhibitors, while transformative, primary, and acquired resistance remain significant challenges, often driven by underlying metabolic adaptations [26]. In addition, a growing body of research is focusing on the impact of metabolic reprogramming, a new hallmark of cancer, marked by the alteration of lipid metabolisms that facilitate rapid proliferation and survival based on the high demand for energy needed for tumors to grow [23, 27]. Cancer cells often change their lipid metabolism pathway [28, 29]. This change in metabolism not only meets the energy and synthesis needs of rapid proliferation but also sets up a TME that speeds up the growth of cancer [30, 31]. Since the liver is the main organ for metabolism, finding a novel treatment or medications that can regulate lipid levels is needed to reach the best functional treatment [32, 33]. The role of ACSLs, especially ACSL3, in lipid metabolism has garnered increasing interest, particularly in the context of cancer biology. ACSL3 is an enzyme that catalyzes the conversion of long‐chain fatty acids (LCFA) into their corresponding acyl‐CoA derivatives, a crucial step in fatty acid metabolism [34, 35]. However, its extra activity can induce the activation of other metabolic processes, increase lipogenesis, and promote the risk of cancers, such as breast cancer (BC) or renal cell carcinoma (ccRCC) [20, 36, 37].

Compelling evidence positions ACSL3 not only as a biomarker but also as a potentially novel therapeutic target. Taken together, our study highlights ACSL3 as a gene associated with lipid metabolism and as a robust negative prognostic marker in HCC. Through systematic analysis, we observed consistently high ACSL3 expression in HCC tissues compared with adjacent normal tissues, as well as in HCC cell lines (HepG2 and HUH7). Our findings also suggest that elevated ACSL3 expression correlates with advanced disease stage, poor survival outcomes, and features of a pro‐tumorigenic immune microenvironment, while also showing strong associations with key oncogenic pathways. For example, high ACSL3 expression was predicted to influence the tumor microenvironment and immune infiltration patterns, including CD8+ T cell dynamics, potentially through correlations with proinflammatory states linked to altered lipid metabolism. Elevated inflammatory cytokines such as IL‐6, IL‐1β, and TNF‐α further complicate this relationship. In other words, while ACSL3 may initially support CD8+ T cell activation, chronic exposure is associated with T cell dysfunction and tumor‐promoting conditions.

**FIGURE 9:**
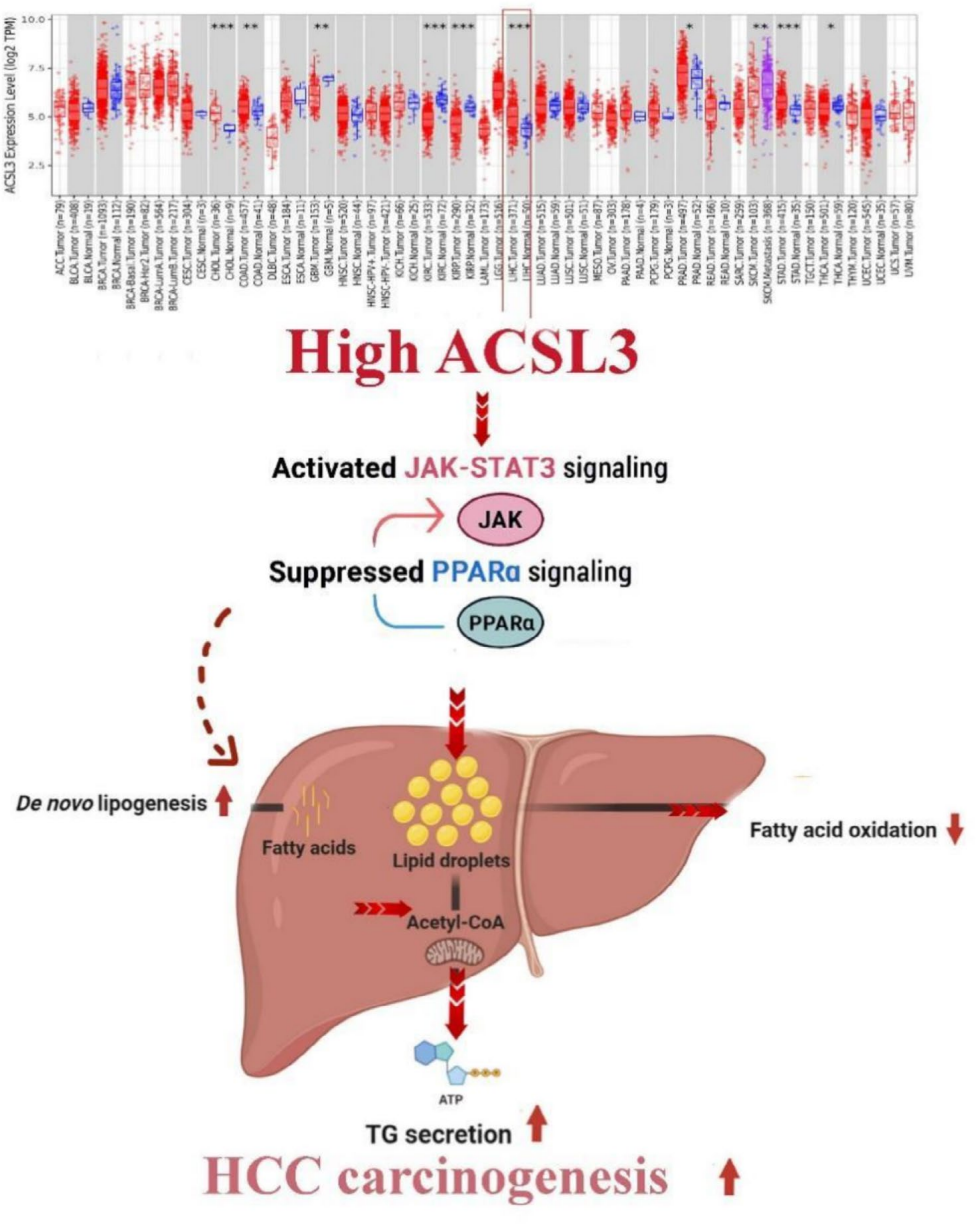
The schematic illustration predicting that high ACSL3 contributes to HCC progression by enhancing JAK/STAT3 activation, which is associated with increased de novo lipogenesis through the upregulation of FASN, SCD, and ACC, creating a self‐reinforcing metabolic loop in which ACSL3 and STAT3 cooperatively promote lipid metabolic rewiring, leading to elevated lipid droplet accumulation and triglyceride secretion. Conversely, high ACSL3 expression is associated with suppression of PPAR‐α signaling, suggesting reduced lipid catabolism in HCC. The computationally inferred pathways are indicated with dashed lines to distinguish them from experimentally supported interactions.

Gene set enrichment (GSEA) and KEGG pathway analyses further predict that ACSL3 overexpression may contribute to a lipid‐enriched microenvironment associated with STAT3 activation. In turn, STAT3 signaling shows correlative upregulation of lipogenic enzymes (FASN, SCD, ACC), suggesting a feedforward loop in which ACSL3‐linked lipid metabolism could reinforce STAT3‐driven lipogenesis. These associations position ACSL3 as a potential contributor to metabolic reprogramming in HCC progression (Figure 9). A particularly promising avenue, based on correlative data, lies in combining ACSL3‐targeted strategies with inhibitors of other lipogenic enzymes. Our analysis revealed strong positive correlations between ACSL3 and de novo lipogenesis (DNL) enzymes such as FASN and ACC, supporting the hypothesis of a coordinated pathway driving lipid accumulation.

Despite these predictive insights, several challenges remain. First, our model suggests that ACSL3 expression correlates with modulation of the JAK/STAT signaling pathway and PD‐L1 expression. While ACSL3 knockdown was associated with reduced STAT3 phosphorylation, the specific lipid mediators underlying this correlation remain unidentified. The absence of comprehensive lipidomic data is a key limitation, and future studies employing untargeted lipidomics in ACSL3‐manipulated cells will be critical to validate these predictions. Second, to our knowledge, this study provides among the first correlative evidence linking ACSL3 with PD‐L1 regulation. Although lipid metabolism and immune checkpoint expression are increasingly connected in cancer biology, the direct mechanistic pathway from ACSL3 to PD‐L1 remains to be established. Future experiments such as kinase activity assays, RNA pulldown, and protein stability assays will be essential to determine whether ACSL3 influences PD‐L1 primarily through upstream kinase activity, transcript stabilization, protein turnover, or other mechanisms yet to be discovered.

By addressing these limitations, future research can move beyond predictive correlations to mechanistic validation, ultimately clarifying the lipid‐driven axis of immune evasion associated with ACSL3. Such work may identify novel therapeutic opportunities to overcome checkpoint inhibitor resistance in HCC and potentially other cancers.

## 5 Conclusion

The oncogenic role of ACSL3 in HCC development underscores its pivotal contribution to metabolic reprogramming and tumor progression by activating lipid biosynthesis signaling pathways, such as the JAK/STAT signaling pathway activity, through lipid metabolites or downstream signaling cascades that promote STAT3 phosphorylation to enhance tumor survival, proliferation, and immune evasion, creating a feed‐forward loop that exacerbates HCC aggressiveness. In addition, ACSL3 suppresses beta‐oxidation, redirecting fatty acids toward storage as lipid droplets rather than mitochondrial energy production, thereby fostering a lipid‐rich microenvironment conducive to tumor growth. Therefore, targeting ACSL3 presents a promising therapeutic strategy for HCC management. However, future research should prioritize biomarker‐driven clinical trials and combinatorial regimens to maximize the therapeutic impact of ACSL3 inhibition in HCC.

## Author Contributions

**Melika Amelimojarad and Mandana Amelimojarad:** conceptualization, writing – original draft, writing – review and editing. **Alireza Pourmahdian:** formal analysis. **Zhang Lu:** investigation. All authors: writing – review and editing.

## Funding

The authors have nothing to report.

## Ethics Statement

The authors have nothing to report.

## Consent

The authors have nothing to report.

## Conflicts of Interest

The authors declare no conflicts of interest.

## Data Availability

The data that support the findings of this study are available on request from the corresponding author. The data are not publicly available due to privacy or ethical restrictions.
